# Genome sequence of the *Lebeckia ambigua*-nodulating “*Burkholderia sprentiae”* strain WSM5005^T^

**DOI:** 10.4056/sigs.4558268

**Published:** 2013-12-15

**Authors:** Wayne Reeve, Sofie De Meyer, Jason Terpolilli, Vanessa Melino, Julie Ardley, Tian Rui, Ravi Tiwari, John Howieson, Ron Yates, Graham O’Hara, Megan Lu, David Bruce, Chris Detter, Roxanne Tapia, Cliff Han, Chia-Lin Wei, Marcel Huntemann, James Han, I-Min Chen, Konstantinos Mavromatis, Victor Markowitz, Ernest Szeto, Natalia Ivanova, Natalia Mikhailova, Galina Ovchinnikova, Ioanna Pagani, Amrita Pati, Lynne Goodwin, Lin Peters, Sam Pitluck, Tanja Woyke, Nikos Kyrpides

**Affiliations:** 1 Centre for Rhizobium Studies, Murdoch University, Western Australia, Australia; 2 Department of Agriculture and Food, Western Australia, Australia; 3 DOE Joint Genome Institute, Walnut Creek, California, USA; 4 Los Alamos National Laboratory, Bioscience Division, Los Alamos, New Mexico, USA; 5 Biological Data Management and Technology Center, Lawrence Berkeley National Laboratory, Berkeley, California, USA

**Keywords:** root-nodule bacteria, nitrogen fixation, rhizobia, *Alphaproteobacteria*

## Abstract

*“Burkholderia sprentiae”* strain WSM5005^T^ is an aerobic, motile, Gram-negative, non-spore-forming rod that was isolated in Australia from an effective N_2_-fixing root nodule of *Lebeckia ambigua* collected in Klawer, Western Cape of South Africa, in October 2007. Here we describe the features of *“Burkholderia sprentiae”* strain WSM5005^T^, together with the genome sequence and its annotation. The 7,761,063 bp high-quality-draft genome is arranged in 8 scaffolds of 236 contigs, contains 7,147 protein-coding genes and 76 RNA-only encoding genes, and is one of 20 rhizobial genomes sequenced as part of the DOE Joint Genome Institute 2010 Community Sequencing Program.

## Introduction

Legumes of the *Fabaceae* family of flowering plants have the unique capacity to form a symbiotic N_2_-fixing symbiosis with soil-inhabiting root nodule bacteria (RNB). This symbiosis supplies leguminous species with the essential bioavailable nitrogen that could otherwise not be obtained from soils that are inherently infertile. The agricultural region of south-west Western Australia contains such impoverished soils and the successful establishment of effective legume-RNB symbioses has been exploited to drive plant and animal productivity in this landscape without the reliance on nitrogenous fertilizer [1,2]. This landscape’s rainfall patterns appear to be changing, from a dry Mediterranean-type distribution to a generally reduced annual rainfall with a less predictable distribution [3]. Due to changes in rainfall patterns, the reproduction of the commercially used annual legume species is challenged. Perennial species might be more able to adapt to climate change, though few commercial perennial forage legumes are adapted to the acid and infertile soils encountered in the region [2]. Therefore, deep-rooted herbaceous perennial legumes including *Rhynchosia* and *Lebeckia* species adapted to acid and infertile soils have been investigated for use in this Australian agricultural setting [2,4,5]. The genus *Lebeckia* Thunb. is part of the *Crotalarieae* tribe, and refers to a group of 33 species of papilionoid legumes that are endemic to the southern and western parts of South Africa, which have similar soil and climate conditions to Western Australia [6,7]. This genus has recently been revised and is now subdivided into several sections, including *Lebeckia* s.s., *Calobota* and *Wiborgiella* [7]. The *Lebeckia* s.s. section, which includes *L. ambigua*, can easily be distinguished from other species by their acicular leaves and 5+5 anther arrangement [7-9].

In four expeditions to the Western Cape of South Africa, between 2002 and 2007, nodules and seeds of *Lebeckia ambigua* were collected and stored [5]. The isolation of RNB from these nodules gave rise to a collection of 23 microsymbionts that clustered into five groups within the genus *Burkholderia* [5]. Unlike most of the previously studied rhizobial *Burkholderia* strains, this South African group appears to be associated with papilionoid forage legumes (rather than *Mimosa* spp.). One of these *Burkholderia* strains has now been designated as the type strain of the new species *“Burkholderia sprentiae”* strain WSM5005^T^ [10]. This isolate effectively nodulates *Lebeckia ambigua* and *L. sepiaria* [5]. Here we present a summary classification and a set of general features for *“Burkholderia sprentiae”* strain WSM5005^T^ together with the description of the complete genome sequence and its annotation.

## Classification and general features

**Figure 1:**
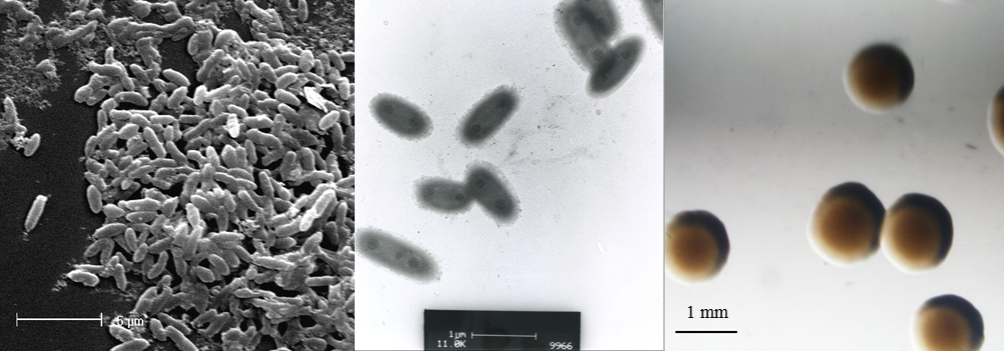
Images of *“Burkholderia sprentiae”* strain WSM5005^T^ using scanning (Left) and transmission (Center) electron microscopy and the colony morphology on a solid medium (Right).

*“Burkholderia sprentiae”* strain WSM5005^T^ is a motile, Gram-negative, non-spore-forming rod (Figure 1, left and center panels) in the order *Burkholderiales* of the class *Betaproteobacteria* [10]. It is fast growing, forming 2-4 mm diameter colonies within 2-3 days when grown on half Lupin Agar (½LA) [11] at 28°C. Colonies on ½LA are white-opaque, slightly domed, moderately mucoid with smooth margins (Figure 1, right panel).

**Figure 2:**
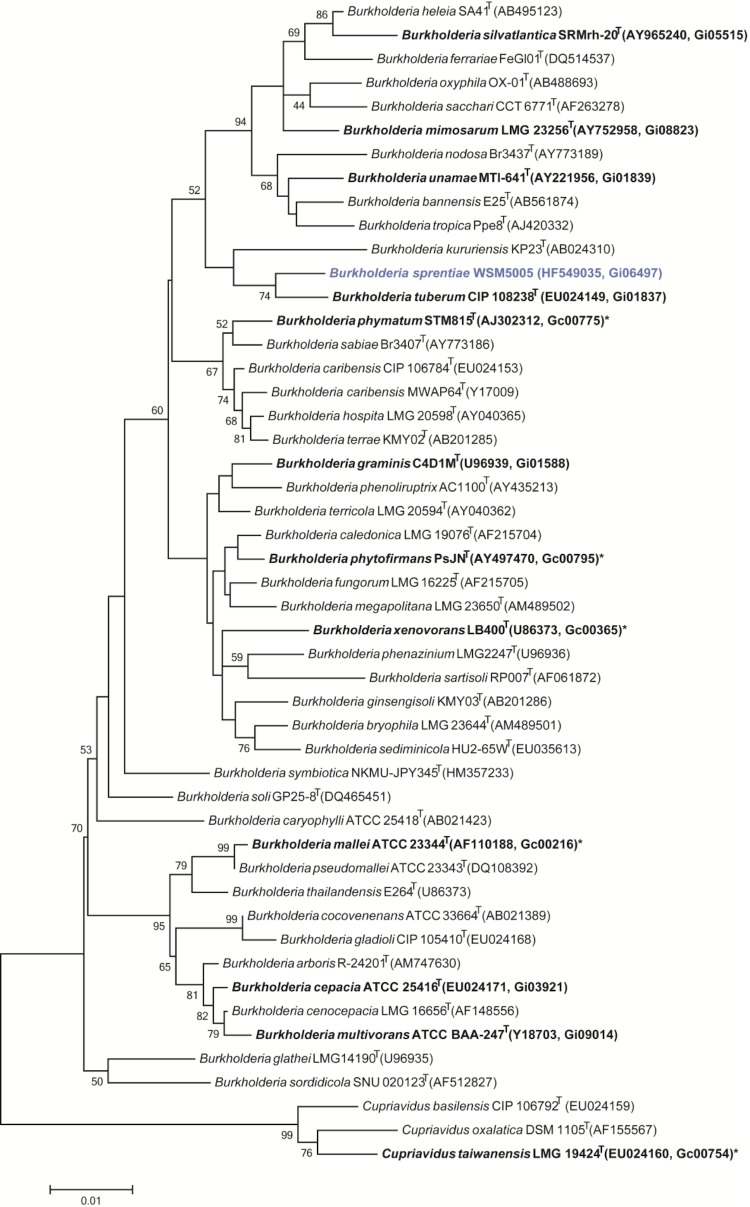
Phylogenetic tree showing the relationships of *“Burkholderia sprentiae”* strain WSM5005^T^ (shown in blue print) with some of the bacteria in the order *Burkholderiales* based on aligned sequences of the 16S rRNA gene (1,322 bp internal region). All sites were informative and there were no gap-containing sites. Phylogenetic analyses were performed using MEGA, version 5.05 [25]. The tree was built using the maximum likelihood method with the General Time Reversible model. Bootstrap analysis [26] with 500 replicates was performed to assess the support of the clusters. Type strains are indicated with a superscript T. Strains with a genome sequencing project registered in GOLD [27] are in bold print and the GOLD ID is mentioned after the accession number. Published genomes are designated with an asterisk.

Minimum Information about the Genome Sequence (MIGS) is provided in Table 1. Figure 2 shows the phylogenetic relationship of *“Burkholderia sprentiae”* strain WSM5005^T^ in a 16S rRNA sequence based tree. This strain clusters closest to *Burkholderia tuberum* STM678^T^ (CIP 108238^T^) and *Burkholderia kururiensis* KP23^T^ with 98.2% and 96.9% sequence identity, respectively.

**Table 1:** Classification and general features of *“Burkholderia sprentiae”* strain WSM5005^T^ according to the MIGS recommendations [12,13].

**MIGS ID**	**Property**	**Term**	**Evidence code**
	Current classification	Domain *Bacteria*	TAS [13]
		Phylum *Proteobacteria*	TAS [14]
		Class *Betaproteobacteria*	TAS [15,16]
		Order *Burkholderiales*	TAS [15,17]
		Family *Burkholderiaceae*	TAS [15,18]
		Genus *Burkholderia*	TAS [19-21]
		Species *“Burkholderia sprentiae”*	TAS [10]
	
	Gram stain	Negative	IDA [22]
	Cell shape	Rod	IDA
	Motility	Motile	IDA
	Sporulation	Non-sporulating	IDA [22]
	Temperature range	Mesophile	IDA [22]
	Optimum temperature	28°C	IDA
	Salinity	Not reported	
MIGS-22	Oxygen requirement	Aerobic	IDA
	Carbon source	Not reported	
	Energy source	Chemoorganotroph	IDA [22]
MIGS-6	Habitat	Soil, root nodule on host	IDA
MIGS-15	Biotic relationship	Free living, symbiotic	IDA
MIGS-14	Pathogenicity	Non-pathogenic	NAS
	Biosafety level	1	TAS [23]
	Isolation	Root nodule	IDA
MIGS-4	Geographic location	South Africa	IDA
MIGS-5	Nodule collection date	October, 2007	IDA
MIGS-4.1	Longitude	18.621111	IDA
MIGS-4.2	Latitude	-31.799722	IDA
MIGS-4.3	Depth	Not recorded	
MIGS-4.4	Altitude	Not recorded	

Evidence codes – IDA: Inferred from Direct Assay; TAS: Traceable Author Statement (i.e., a direct report exists in the literature); NAS: Non-traceable Author Statement (i.e., not directly observed for the living, isolated sample, but based on a generally accepted property for the species, or anecdotal evidence). These evidence codes are from the Gene Ontology project [24].

### Symbiotaxonomy

*“Burkholderia sprentiae”* strain WSM5005^T^ is part of a cadre of *Burkholderia* strains that were assessed for nodulation and nitrogen fixation on three separate *L. ambigua* genotypes (CRSLAM-37, CRSLAM-39 and CRSLAM-41) and on *L. sepiaria* [5]. Representatives of this group of nodule bacteria are generally Nod^+^ and Fix^-^ on *Macroptillium atropurpureum* and appear to have a very narrow host range for symbiosis. They belong to a group of *Burkholderia* strains that nodulate papilionoid forage legumes rather than the classical *Burkholderia* hosts *Mimosa* spp. (*Mimosoideae*) [28].

## Genome sequencing and annotation information

### Genome project history

This organism was selected for sequencing on the basis of its environmental and agricultural relevance to issues in global carbon cycling, alternative energy production, and biogeochemical importance, and is part of the Community Sequencing Program at the U.S. Department of Energy, Joint Genome Institute (JGI) for projects of relevance to agency missions. The genome project is deposited in the Genomes OnLine Database [27] and an improved-high-quality-draft genome sequence in IMG. Sequencing, finishing and annotation were performed by the JGI. A summary of the project information is shown in Table 2.

**Table 2:** Genome sequencing project information for *“Burkholderia sprentiae”* strain WSM5005^T^

**MIGS ID**	**Property**	**Term**
MIGS-31	Finishing quality	Improved high-quality draft
MIGS-28	Libraries used	Illumina GAii shotgun and paired end 454 libraries
MIGS-29	Sequencing platforms	Illumina HiSeq 2000 and 454 GS FLX Titanium technologies
MIGS-31.2	Sequencing coverage	8.4x 454 paired end, 300 x Illumina
MIGS-30	Assemblers	VELVET 1.013, Newbler 2.3, phrap 4.24
MIGS-32	Gene calling methods	Prodigal 1.4, GenePRIMP
	GOLD ID	Gi06497
	GenBank ID	AXBN01000000
	Database: IMG	2510065045
	Project relevance	Symbiotic N_2_fixation, agriculture

### Growth conditions and DNA isolation

*“Burkholderia sprentiae”* strain WSM5005^T^ was grown to mid logarithmic phase in TY rich medium [29] on a gyratory shaker at 28°C. DNA was isolated from 60 mL of cells using a CTAB (Cetyl trimethyl ammonium bromide) bacterial genomic DNA isolation method [30].

### Genome sequencing and assembly

The genome of *“Burkholderia sprentiae”* strain WSM5005^T^ was sequenced at the Joint Genome Institute (JGI) using a combination of Illumina [31] and 454 technologies [32]. An Illumina GAii shotgun library which generated 76,247,610 reads totaling 5,794.8 Mb, and a paired end 454 library with an average insert size of 13 kb which generated 612,483 reads totaling 112.9 Mb of 454 data were generated for this genome. All general aspects of library construction and sequencing performed at the JGI can be found at [30]. The initial draft assembly contained 420 contigs in 8 scaffolds. The 454 paired end data was assembled with Newbler, version 2.3. The Newbler consensus sequences were computationally shredded into 2 kb overlapping fake reads (shreds). Illumina sequencing data were assembled with VELVET, version 1.0.13 [33], and the consensus sequences were computationally shredded into 1.5 kb overlapping fake reads (shreds). We integrated the 454 Newbler consensus shreds, the Illumina VELVET consensus shreds and the read pairs in the 454 paired end library using parallel phrap, version SPS - 4.24 (High Performance Software, LLC). The software Consed [34-36] was used in the following finishing process. Illumina data was used to correct potential base errors and increase consensus quality using the software Polisher developed at JGI (Alla Lapidus, unpublished). Possible mis-assemblies were corrected using gapResolution (Cliff Han, unpublished), Dupfinisher [37], or sequencing cloned bridging PCR fragments with subcloning. Gaps between contigs were closed by editing in Consed, by PCR and by Bubble PCR (J-F Cheng, unpublished) primer walks. A total of 352 additional reactions were necessary to close gaps and to raise the quality of the finished sequence. The estimated genome size is 7.8 Mb and the final assembly is based on 65.2 Mb of 454 draft data which provides an average 8.4× coverage of the genome and 2,340 Mb of Illumina draft data which provides an average 300× coverage of the genome.

### Genome annotation

Genes were identified using Prodigal [38] as part of the DOE-JGI Annotation pipeline [39], followed by a round of manual curation using the JGI GenePRIMP pipeline [40]. The predicted CDSs were translated and used to search the National Center for Biotechnology Information (NCBI) non-redundant database, UniProt, TIGRFam, Pfam, PRIAM, KEGG, COG, and InterPro databases. These data sources were combined to assert a product description for each predicted protein. Non-coding genes and miscellaneous features were predicted using tRNAscan-SE [41], RNAMMer [42], Rfam [43], TMHMM [44], and SignalP [45]. Additional gene prediction analyses and functional annotation were performed within the Integrated Microbial Genomes (IMG-ER) platform [46].

## Genome properties

**Figure 3:**
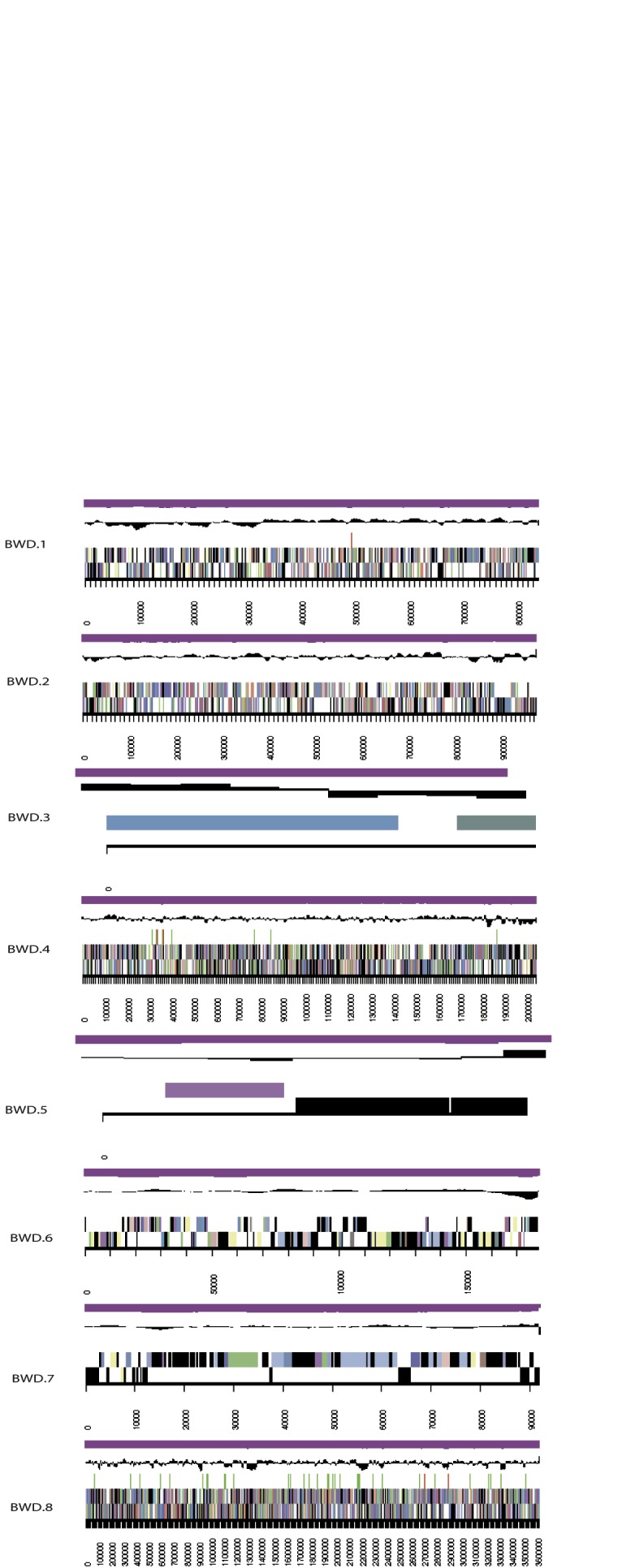
Graphical map of the chromosome of *“Burkholderia sprentiae”* strain WSM5005^T^. From the bottom to the top of each scaffold: Genes on forward strand (color by COG categories as denoted by the IMG platform), Genes on reverse strand (color by COG categories), RNA genes (tRNAs green, sRNAs red, other RNAs black), GC content, GC skew.

**Figure 4:**
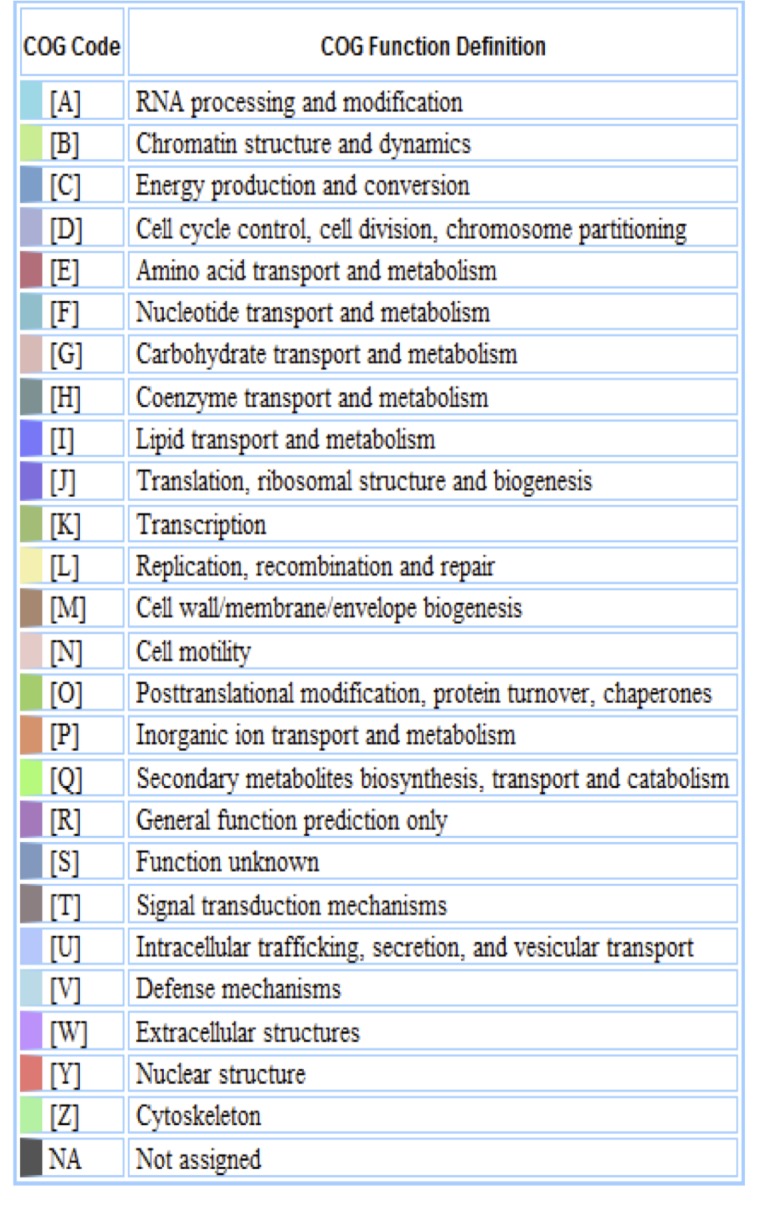
Color code for Figure 3.

The genome is 7,761,063 nucleotides with 63.18% GC content (Table 3) and comprised of 8 scaffolds of 236 contigs. From a total of 7,223 genes, 7,147 were protein encoding and 76 RNA only encoding genes. Within the genome, 377 pseudogenes were also identified. The majority of genes (76.16%) were assigned a putative function whilst the remaining genes were annotated as hypothetical. The distribution of genes into COGs functional categories is presented in Table 4, Figure 3 and Figure 4.

**Table 3:** Genome Statistics for *“Burkholderia sprentiae”* strain WSM5005^T^.

**Attribute**	**Value**	**% of Total**
Genome size (bp)	7,761,063	100
DNA coding region (bp)	6,514,546	83.94
DNA G+C content (bp)	4,903,511	63.18
Number of scaffolds	8	
Number of contigs	236	
Total genes	7,223	100
RNA genes	76	1.05
Protein-coding genes	7,147	98.95
Genes with function prediction	5,501	76.16
Genes assigned to COGs	5,456	75.54
Genes assigned Pfam domains	5,800	80.30
Genes with signal peptides	687	9.51
Genes with transmembrane helices	1,634	22.62
CRISPR repeats	0	

**Table 4:** Number of protein coding genes of *“Burkholderia sprentiae”* strain WSM5005^T^ associated with the general COG functional categories.

Code	Value	%age	Description
J	205	3.34	Translation, ribosomal structure and biogenesis
A	2	0.03	RNA processing and modification
K	566	9.22	Transcription
L	257	4.18	Replication, recombination and repair
B	1	0.02	Chromatin structure and dynamics
D	46	0.75	Cell cycle control, mitosis and meiosis
Y	0	0.00	Nuclear structure
V	70	1.14	Defense mechanisms
T	313	5.10	Signal transduction mechanisms
M	409	6.66	Cell wall/membrane biogenesis
N	114	1.86	Cell motility
Z	0	0.00	Cytoskeleton
W	2	0.03	Extracellular structures
U	154	2.51	Intracellular trafficking and secretion
O	185	3.01	Posttranslational modification, protein turnover, chaperones
C	442	7.20	Energy production conversion
G	486	7.91	Carbohydrate transport and metabolism
E	576	9.38	Amino acid transport metabolism
F	96	1.56	Nucleotide transport and metabolism
H	219	3.57	Coenzyme transport and metabolism
I	288	4.69	Lipid transport and metabolism
P	282	4.59	Inorganic ion transport and metabolism
Q	176	2.87	Secondary metabolite biosynthesis, transport and catabolism
R	738	12.02	General function prediction only
S	515	8.38	Function unknown
-	1,767	24.46	Not in COGS
